# 2,4-Di-*tert*-butyl-6-[(2,5-difluorophenyl)iminomethyl]phenol

**DOI:** 10.1107/S1600536809041099

**Published:** 2009-10-17

**Authors:** Ömer Çelik, Veli T. Kasumov, Ertan Şahin

**Affiliations:** a Department of Physics, Faculty of Science and Art, Harran University, 63300 Şanlıurfa, Turkey; b Department of Chemistry, Faculty of Science and Art, Harran University, 63300 Şanlıurfa, Turkey; c Department of Chemistry, Faculty of Science and Art, Atatürk University, 25100 Erzurum, Turkey

## Abstract

In the title Schiff base, C_21_H_25_F_2_NO, the dihedral angle between the aromatic rings is 27.90(5)° and an intramolecular O—H⋯N hydrogen bond occurs. In the crystal, the molecules are linked by C—H⋯O, C—H⋯N and C—H⋯F interactions.

## Related literature

For background on the photochromic behavior of salicyli­dene­anilines, see: Brown (1971 ▶); Chemla & Zyss (1987 ▶); MacDonald & Whitesides (1994 ▶); Cohen *et al.* (1966 ▶). For related compounds, see: Ancın *et al.* (2007 ▶); Kasumov, Köksal & Köseoĝlu (2004 ▶); Kasumov, Medjidov, Ya­ylı & Zeren (2004 ▶); Çelik *et al.* (2007 ▶, 2009 ▶). For graph-set notation, see: Bernstein *et al.* (1995 ▶); Etter (1991 ▶).  
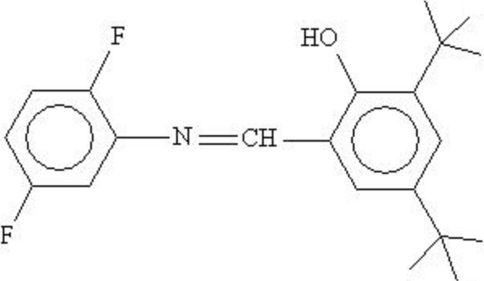


## Experimental

### 

#### Crystal data

C_21_H_25_F_2_NO
*M* *_r_* = 345.42
Monoclinic, 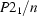

*a* = 6.423 (5) Å
*b* = 17.386 (5) Å
*c* = 17.337 (5) Å
β = 90.319 (5)°
*V* = 1936.0 (17) Å^3^
*Z* = 4
Mo *K*α radiation
μ = 0.09 mm^−1^
*T* = 293 K
0.20 × 0.20 × 0.20 mm

#### Data collection

Rigaku Rxdiffractometer
Absorption correction: multi-scan (Blessing, 1995 ▶) *T*_min_ = 0.983, *T*_max_ = 0.983
50854 measured reflections
5662 independent reflections
2715 reflections with *I* > 2σ(*I*)
*R*_int_ = 0.099

#### Refinement

*R*[*F* ^2^ > 2σ(*F* ^2^)] = 0.057
*wR*(*F* ^2^) = 0.144
*S* = 0.96
5662 reflections
238 parameters
H atoms treated by a mixture of independent and constrained refinement
Δρ_max_ = 0.11 e Å^−3^
Δρ_min_ = −0.13 e Å^−3^

### 

Data collection: *CrystalClear* (Rigaku/MSC, 2005 ▶); cell refinement: *CrystalClear*; data reduction: *CrystalClear*; program(s) used to solve structure: *SHELXS97* (Sheldrick, 2008 ▶); program(s) used to refine structure: *SHELXL97* (Sheldrick, 2008 ▶); molecular graphics: *ORTEP-3* (Farrugia, 1997 ▶); software used to prepare material for publication: *SHELXTL* (Sheldrick, 2008 ▶) and local programs.

## Supplementary Material

Crystal structure: contains datablocks I, global. DOI: 10.1107/S1600536809041099/jh2098sup1.cif

Structure factors: contains datablocks I. DOI: 10.1107/S1600536809041099/jh2098Isup2.hkl

Additional supplementary materials:  crystallographic information; 3D view; checkCIF report

## Figures and Tables

**Table 1:** Hydrogen-bond geometry (Å, °)

*D*—H⋯*A*	*D*—H	H⋯*A*	*D*⋯*A*	*D*—H⋯*A*
O—H0⋯N	0.82	1.88	2.615 (2)	149
C16—H16*A*⋯O	0.96	2.27	2.935 (3)	126
C15—H15*A*⋯O	0.96	2.40	3.038 (2)	123
C16—H16*C*⋯N^i^	0.96	2.72	3.643 (3)	162
C21—H21*B*⋯F2^ii^	0.96	2.68	3.498 (3)	143

Symmetry codes: (i) 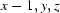; (ii) 
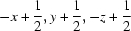
.

**Fig. 1.**
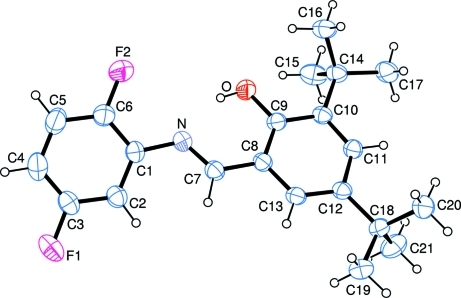
ORTEP III diagram of the compound, showing the molecular numbering scheme. Displacement ellipsoids are drawn at 50% probability for all atoms except H.

**Fig. 2.**
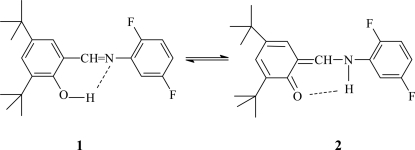
Tautomerism in the title compound.

## supplementary crystallographic information

### Comment

Proton tautomerization plays an important role in many fields of chemistry and biochemistry. The tautomerization in salicylideneanilines has been the subject of particular interest, because it is closely related to thermochromisim and photochromisim. While salicylideneanilines are widely used as a precursor compounds for a design of various type new metal complexes they are also a convenient model compounds for studying theoretical aspects of coordination chemistry and  photochemistry,  as well as for designing molecular architecture by means of molecular motifs capable of H-bond formation. The existence of photochromic behavior suggests the possibility of using these compounds as elements for constructing the optical switches or optical memory devices (Brown, 1971; Chemla *et al.*, 1987; Cohen *et al.*, 1966; Chemla, *et al.*, 1987; MacDonald, *et al.*, 1994). As part of our interest on electron transfer and complexation behaviors of redox-active salicylaldimines, obtained from bulky di-tertbutylated sterically hindered aminophenols,  salicylaldehydes and aryl amines and their complexes (Kasumov *et al.*, 2004; Kasumov *et al.*, 2004), we decided to prepare and structurally investigate the bidentate salicylaldimines derived from 3,5-di-tert-butyl-salicylaldehyde and difluorinated anilines.

In the compound, the difluoroaniline atoms (P1) and benzylidene atoms (P2) are plane and the dihedral angle between P1 and P2 planes is 27.90 (5)°. The maximum deviations from the P1 plane of  C3 and P2 plane of C11 are -0.008 Å and 0.002 Å, respectivity. The bond between N and C7 atoms is double bond, whose length is  1.282 (2)Å, and the conformation at this double bond is trans with the torsion angle C1-N-C7-C8 is 178.2 (1)°. The bond lengths are as expected. Similar results are were observed in the study of N-[5- methylisoxazole-amino-3-yl]-3, 5-di-tert-butylsalicylaldimine amino-3-yl]-3,5 -di-tert-butylsalicylaldimine(Çelik *et al.*, 2007), N-[1-(3-Aminopropyl) imidazole]-3,5-di-tertbutylsalicylaldimine(Çelik *et al.*, 2009) and N,N'-bis- (5-methylsalicylidene)-2,2'-diamino-4-4'-di-(trifloromethyl)-diphenyl disulfide (Ancın, *et al.*, 2007).

In Table 2 is given interactions have types of O-H···N, C-H···O, C-H···N and C-H···F. O-H···N hydrogen bond which are intramolecular interaction causes to reversible proton transfer between imine N atom and the hydroxyl O atom. The three intra-molecular interaction have strong effects for molecule. C-H···O hydrogen bond which are intramolecular interaction causes to reversible proton transfer between methyl C atom and the hydroxyl O atom. Similar Schiff bases usually show photochromism and thermocromism because of the above mentioned intramolecular hydrogen bonds. Similar proton transfer has nor been determined in the molecular structure of our compound. The cause of this results may be explained by a steric effect and are effective for the molecular packing of the compound.

### Experimental

The pale yellow crystalline the title compound was prepared by using standard procedure involving the condensation of equimolar amount of 3,5-di-*tert*-butyl-2-hydroxybenzaldehyde with 2,5 difluoroaniline in refluxing ethanol in the presence of catalytic amount of formic acid (3–4 drops). To a stirred and heated (60 °C) solution of 3,5-di-*tert*-butyl-2-hydroxybenzaldehyde (0.936 g, 4 mmol) in absolute ethanol (80 ml), a solution of 0.516 g (4 mmol) of 2,5-difluoroaniline in 5 ml methanol was added immediately. Then 4 drops of formic acid was added to this solution and refluxed for 24 h. The volume of the reaction mixture was evaporated to 25 ml and after cooling to 15 °C, the yellow crystals were collected and air dried to yield 1.298 g (94%).

### Crystal data

**Table:**

C_21_H_25_F_2_NO	*F*(000) = 736
*M**_r_* = 345.42	*D*_x_ = 1.185 Mg m^−^^3^
Monoclinic, *P*2_1_/*n*	Mo *K*α radiation, λ = 0.71073 Å
*a* = 6.423 (5) Å	Cell parameters from 5929 reflections
*b* = 17.386 (5) Å	θ = 2.6–30.5°
*c* = 17.337 (5) Å	µ = 0.09 mm^−^^1^
β = 90.319 (5)°	*T* = 293 K
*V* = 1936.0 (17) Å^3^	Needle, pale yellow
*Z* = 4	0.20 × 0.20 × 0.20 mm

### Data collection

**Table:**

Rigaku Rx diffractometer	5662 independent reflections
Radiation source: fine-focus sealed tube	2715 reflections with *I* > 2σ(*I*)
graphite	*R*_int_ = 0.099
Detector resolution: 10.0000 pixels mm^-1^	θ_max_ = 30.5°, θ_min_ = 2.6°
dtprofit.ref scans	*h* = −9→7
Absorption correction: multi-scan (Blessing, 1995)	*k* = −24→24
*T*_min_ = 0.983, *T*_max_ = 0.983	*l* = −24→24
50854 measured reflections	

### Refinement

**Table:**

Refinement on *F*^2^	Primary atom site location: structure-invariant direct methods
Least-squares matrix: full	Secondary atom site location: difference Fourier map
*R*[*F*^2^ > 2σ(*F*^2^)] = 0.057	Hydrogen site location: mixed
*wR*(*F*^2^) = 0.144	H atoms treated by a mixture of independent and constrained refinement
*S* = 0.96	*w* = 1/[σ^2^(*F*_o_^2^) + (0.0514*P*)^2^] where *P* = (*F*_o_^2^ + 2*F*_c_^2^)/3
5662 reflections	(Δ/σ)_max_ < 0.001
238 parameters	Δρ_max_ = 0.11 e Å^−^^3^
0 restraints	Δρ_min_ = −0.13 e Å^−^^3^
none constraints	

### Special details

**Table:**

Geometry. All e.s.d.'s (except the e.s.d. in the dihedral angle between two l.s. planes) are estimated using the full covariance matrix. The cell e.s.d.'s are taken into account individually in the estimation of e.s.d.'s in distances, angles and torsion angles; correlations between e.s.d.'s in cell parameters are only used when they are defined by crystal symmetry. An approximate (isotropic) treatment of cell e.s.d.'s is used for estimating e.s.d.'s involving l.s. planes.
Refinement. Refinement of *F*^2^ against ALL reflections. The weighted *R*-factor *wR* and goodness of fit *S* are based on *F*^2^, conventional *R*-factors *R* are based on *F*, with *F* set to zero for negative *F*^2^. The threshold expression of *F*^2^ > σ(*F*^2^) is used only for calculating *R*-factors(gt) *etc*. and is not relevant to the choice of reflections for refinement. *R*-factors based on *F*^2^ are statistically about twice as large as those based on *F*, and *R*- factors based on ALL data will be even larger.

### Fractional atomic coordinates and isotropic or equivalent isotropic displacement parameters (Å^2^)

**Table:**

	*x*	*y*	*z*	*U*_iso_*/*U*_eq_	
F1	1.34032 (17)	−0.06161 (7)	0.45693 (7)	0.0928 (4)	
F2	0.7822 (2)	−0.18169 (7)	0.25642 (7)	0.1106 (5)	
O	0.43585 (19)	−0.00558 (7)	0.19801 (7)	0.0716 (3)	
N	0.7118 (2)	−0.03731 (8)	0.30481 (8)	0.0599 (3)	
C1	0.8873 (2)	−0.07788 (9)	0.33323 (9)	0.0579 (4)	
C2	1.0326 (3)	−0.04776 (11)	0.38404 (10)	0.0632 (4)	
C3	1.2008 (3)	−0.09201 (11)	0.40566 (10)	0.0670 (4)	
C4	1.2353 (3)	−0.16381 (13)	0.37793 (11)	0.0817 (6)	
C5	1.0919 (3)	−0.19439 (12)	0.32701 (12)	0.0887 (6)	
C6	0.9227 (3)	−0.15091 (11)	0.30581 (10)	0.0727 (5)	
C7	0.6290 (3)	0.01624 (9)	0.34520 (10)	0.0571 (4)	
C8	0.4535 (2)	0.06101 (9)	0.31824 (8)	0.0533 (4)	
C9	0.3601 (2)	0.04900 (8)	0.24564 (9)	0.0542 (4)	
C10	0.1864 (2)	0.09270 (8)	0.22268 (8)	0.0531 (4)	
C11	0.1149 (3)	0.14730 (9)	0.27431 (9)	0.0551 (4)	
C12	0.2021 (2)	0.16151 (8)	0.34774 (9)	0.0517 (4)	
C13	0.3714 (2)	0.11717 (8)	0.36766 (9)	0.0543 (4)	
C14	0.0847 (3)	0.08127 (9)	0.14289 (9)	0.0608 (4)	
C15	0.2440 (3)	0.10244 (12)	0.07964 (10)	0.0878 (6)	
C16	0.0150 (3)	−0.00280 (10)	0.13124 (10)	0.0745 (5)	
C17	−0.1094 (3)	0.13131 (11)	0.13211 (11)	0.0852 (6)	
C18	0.1136 (3)	0.22420 (9)	0.40007 (9)	0.0590 (4)	
C19	0.2129 (3)	0.22172 (12)	0.48075 (10)	0.0835 (6)	
C20	−0.1214 (3)	0.21370 (11)	0.40956 (11)	0.0767 (5)	
C21	0.1544 (3)	0.30292 (10)	0.36414 (12)	0.0909 (6)	
H0	0.5354	−0.0269	0.2187	0.107*	
H2	1.018 (3)	0.0018 (10)	0.4049 (10)	0.072 (5)*	
H4	1.3524	−0.1916	0.3929	0.098*	
H5	1.1096	−0.2437	0.3073	0.106*	
H7	0.681 (2)	0.0293 (9)	0.4007 (10)	0.071 (5)*	
H11	0.001 (2)	0.1785 (8)	0.2587 (8)	0.054 (4)*	
H13	0.4337	0.1247	0.4156	0.065*	
H15A	0.3659	0.0709	0.0853	0.132*	
H15B	0.2820	0.1556	0.0846	0.132*	
H15C	0.1830	0.0939	0.0297	0.132*	
H16A	0.1327	−0.0363	0.1374	0.112*	
H16B	−0.0422	−0.0088	0.0803	0.112*	
H16C	−0.0888	−0.0156	0.1687	0.112*	
H17A	−0.1680	0.1223	0.0819	0.128*	
H17B	−0.0719	0.1845	0.1369	0.128*	
H17C	−0.2100	0.1184	0.1708	0.128*	
H19A	0.1544	0.2617	0.5121	0.125*	
H19B	0.3604	0.2292	0.4766	0.125*	
H19C	0.1858	0.1727	0.5041	0.125*	
H20A	−0.1749	0.2537	0.4421	0.115*	
H20B	−0.1487	0.1645	0.4326	0.115*	
H20C	−0.1881	0.2163	0.3599	0.115*	
H21A	0.0982	0.3423	0.3968	0.136*	
H21B	0.0890	0.3055	0.3143	0.136*	
H21C	0.3017	0.3105	0.3588	0.136*	

### Atomic displacement parameters (Å^2^)

**Table:**

	*U*^11^	*U*^22^	*U*^33^	*U*^12^	*U*^13^	*U*^23^
F1	0.0730 (7)	0.1143 (9)	0.0908 (8)	−0.0059 (6)	−0.0254 (6)	0.0194 (6)
F2	0.1241 (10)	0.0941 (9)	0.1131 (10)	0.0261 (7)	−0.0420 (8)	−0.0405 (7)
O	0.0734 (8)	0.0773 (8)	0.0639 (7)	0.0170 (6)	−0.0125 (6)	−0.0166 (6)
N	0.0572 (8)	0.0612 (8)	0.0613 (8)	0.0052 (6)	−0.0076 (6)	−0.0001 (6)
C1	0.0557 (9)	0.0593 (10)	0.0587 (9)	0.0053 (8)	−0.0012 (7)	0.0062 (7)
C2	0.0598 (10)	0.0635 (11)	0.0662 (11)	−0.0011 (8)	−0.0034 (8)	0.0075 (8)
C3	0.0579 (10)	0.0824 (12)	0.0608 (10)	−0.0001 (9)	−0.0042 (8)	0.0134 (9)
C4	0.0724 (12)	0.0961 (15)	0.0766 (13)	0.0274 (11)	0.0037 (10)	0.0123 (11)
C5	0.1007 (16)	0.0829 (13)	0.0825 (13)	0.0350 (12)	−0.0014 (12)	−0.0069 (10)
C6	0.0785 (12)	0.0752 (12)	0.0644 (11)	0.0108 (10)	−0.0086 (9)	−0.0091 (9)
C7	0.0557 (9)	0.0584 (10)	0.0572 (10)	−0.0004 (8)	−0.0077 (8)	0.0019 (7)
C8	0.0518 (9)	0.0542 (9)	0.0538 (9)	−0.0010 (7)	−0.0049 (7)	0.0000 (7)
C9	0.0585 (9)	0.0503 (9)	0.0536 (9)	−0.0021 (7)	−0.0026 (7)	−0.0037 (7)
C10	0.0573 (9)	0.0521 (8)	0.0498 (8)	−0.0045 (7)	−0.0064 (7)	0.0026 (7)
C11	0.0576 (10)	0.0494 (9)	0.0582 (9)	0.0010 (7)	−0.0075 (7)	0.0040 (7)
C12	0.0536 (9)	0.0468 (8)	0.0546 (8)	−0.0063 (7)	−0.0038 (7)	−0.0004 (6)
C13	0.0562 (9)	0.0540 (9)	0.0527 (8)	−0.0049 (7)	−0.0091 (7)	−0.0027 (7)
C14	0.0723 (11)	0.0595 (10)	0.0504 (9)	−0.0034 (8)	−0.0122 (8)	0.0013 (7)
C15	0.1058 (16)	0.0983 (15)	0.0594 (11)	−0.0252 (12)	−0.0046 (11)	0.0053 (10)
C16	0.0868 (13)	0.0699 (11)	0.0667 (11)	−0.0099 (10)	−0.0171 (9)	−0.0068 (8)
C17	0.0960 (15)	0.0814 (13)	0.0779 (13)	0.0107 (11)	−0.0372 (11)	0.0014 (10)
C18	0.0635 (10)	0.0500 (9)	0.0636 (10)	−0.0017 (7)	−0.0001 (8)	−0.0090 (7)
C19	0.0862 (13)	0.0914 (14)	0.0727 (12)	0.0044 (11)	−0.0081 (10)	−0.0281 (10)
C20	0.0649 (11)	0.0869 (13)	0.0784 (12)	0.0017 (9)	0.0046 (9)	−0.0107 (10)
C21	0.1132 (17)	0.0543 (11)	0.1054 (16)	−0.0062 (11)	0.0154 (13)	−0.0068 (10)

### Geometric parameters (Å, °)

**Table:**

F1—C3	1.365 (2)	C2—H2	0.940 (17)
F2—C6	1.351 (2)	C16—H16A	0.9600
O—C9	1.3503 (18)	C16—H16B	0.9600
N—C7	1.282 (2)	C16—H16C	0.9600
N—C1	1.416 (2)	C19—H19A	0.9600
C1—C2	1.383 (2)	C19—H19B	0.9600
C1—C6	1.375 (2)	C19—H19C	0.9600
C3—C4	1.356 (3)	C17—H17A	0.9600
C3—C2	1.376 (2)	C17—H17B	0.9600
C4—C5	1.379 (3)	C17—H17C	0.9600
C6—C5	1.372 (3)	C20—H20A	0.9600
C8—C13	1.404 (2)	C20—H20B	0.9600
C8—C9	1.407 (2)	C20—H20C	0.9600
C8—C7	1.445 (2)	C4—H4	0.9300
C10—C11	1.385 (2)	C15—H15A	0.9600
C10—C9	1.405 (2)	C15—H15B	0.9600
C10—C14	1.540 (2)	C15—H15C	0.9600
C11—C12	1.410 (2)	C5—H5	0.9300
C12—C13	1.375 (2)	C21—H21A	0.9600
C12—C18	1.530 (2)	C21—H21B	0.9600
C14—C17	1.531 (2)	C21—H21C	0.9600
C14—C16	1.542 (2)	C11—H11	0.948 (14)
C14—C15	1.548 (3)	C13—H13	0.9300
C18—C21	1.527 (2)	C7—H7	1.042 (17)
C18—C20	1.530 (3)	O—H0	0.8200
C18—C19	1.535 (2)		
			
C7—N—C1	120.24 (14)	C14—C16—H16C	109.5
C11—C10—C9	116.96 (14)	H16A—C16—H16C	109.5
C11—C10—C14	121.90 (14)	H16B—C16—H16C	109.5
C9—C10—C14	121.13 (14)	C18—C19—H19A	109.5
C10—C11—C12	124.88 (15)	C18—C19—H19B	109.5
C13—C8—C9	119.32 (14)	H19A—C19—H19B	109.5
C13—C8—C7	118.16 (14)	C18—C19—H19C	109.5
C9—C8—C7	122.50 (14)	H19A—C19—H19C	109.5
C13—C12—C11	116.00 (14)	H19B—C19—H19C	109.5
C13—C12—C18	123.12 (14)	C14—C17—H17A	109.5
C11—C12—C18	120.87 (14)	C14—C17—H17B	109.5
C12—C13—C8	122.39 (14)	H17A—C17—H17B	109.5
C6—C1—C2	117.22 (16)	C14—C17—H17C	109.5
C6—C1—N	118.20 (15)	H17A—C17—H17C	109.5
C2—C1—N	124.49 (16)	H17B—C17—H17C	109.5
O—C9—C10	119.66 (14)	C18—C20—H20A	109.5
O—C9—C8	119.88 (14)	C18—C20—H20B	109.5
C10—C9—C8	120.45 (14)	H20A—C20—H20B	109.5
C21—C18—C12	109.36 (14)	C18—C20—H20C	109.5
C21—C18—C20	108.81 (15)	H20A—C20—H20C	109.5
C12—C18—C20	110.42 (13)	H20B—C20—H20C	109.5
C21—C18—C19	109.01 (15)	C3—C4—H4	120.9
C12—C18—C19	111.50 (14)	C5—C4—H4	120.9
C20—C18—C19	107.68 (14)	C14—C15—H15A	109.5
N—C7—C8	122.66 (15)	C14—C15—H15B	109.5
C17—C14—C10	112.19 (14)	H15A—C15—H15B	109.5
C17—C14—C16	106.69 (15)	C14—C15—H15C	109.5
C10—C14—C16	111.20 (13)	H15A—C15—H15C	109.5
C17—C14—C15	108.64 (15)	H15B—C15—H15C	109.5
C10—C14—C15	109.07 (14)	C6—C5—H5	120.5
C16—C14—C15	108.97 (15)	C4—C5—H5	120.5
C4—C3—F1	118.63 (17)	C18—C21—H21A	109.5
C4—C3—C2	123.19 (18)	C18—C21—H21B	109.5
F1—C3—C2	118.17 (18)	H21A—C21—H21B	109.5
C3—C2—C1	119.19 (18)	C18—C21—H21C	109.5
F2—C6—C5	118.48 (17)	H21A—C21—H21C	109.5
F2—C6—C1	118.27 (16)	H21B—C21—H21C	109.5
C5—C6—C1	123.24 (18)	C10—C11—H11	117.7 (9)
C3—C4—C5	118.17 (18)	C12—C11—H11	117.4 (9)
C6—C5—C4	118.96 (19)	C12—C13—H13	118.8
C3—C2—H2	119.2 (11)	C8—C13—H13	118.8
C1—C2—H2	121.6 (11)	N—C7—H7	122.0 (9)
C14—C16—H16A	109.5	C8—C7—H7	115.3 (9)
C14—C16—H16B	109.5	C9—O—H0	109.5
H16A—C16—H16B	109.5		
			
C9—C10—C11—C12	−0.5 (2)	C11—C12—C18—C19	172.11 (15)
C14—C10—C11—C12	−179.12 (14)	C1—N—C7—C8	178.23 (14)
C10—C11—C12—C13	0.3 (2)	C13—C8—C7—N	178.59 (15)
C10—C11—C12—C18	178.97 (15)	C9—C8—C7—N	0.1 (2)
C11—C12—C13—C8	0.0 (2)	C11—C10—C14—C17	−4.9 (2)
C18—C12—C13—C8	−178.57 (14)	C9—C10—C14—C17	176.53 (15)
C9—C8—C13—C12	−0.2 (2)	C11—C10—C14—C16	−124.32 (17)
C7—C8—C13—C12	−178.68 (14)	C9—C10—C14—C16	57.1 (2)
C7—N—C1—C6	155.18 (17)	C11—C10—C14—C15	115.49 (18)
C7—N—C1—C2	−28.3 (2)	C9—C10—C14—C15	−63.06 (19)
C11—C10—C9—O	179.42 (14)	C4—C3—C2—C1	1.8 (3)
C14—C10—C9—O	−2.0 (2)	F1—C3—C2—C1	−178.84 (14)
C11—C10—C9—C8	0.3 (2)	C6—C1—C2—C3	−1.3 (2)
C14—C10—C9—C8	178.94 (14)	N—C1—C2—C3	−177.87 (15)
C13—C8—C9—O	−179.09 (14)	C2—C1—C6—F2	179.66 (16)
C7—C8—C9—O	−0.7 (2)	N—C1—C6—F2	−3.5 (3)
C13—C8—C9—C10	0.0 (2)	C2—C1—C6—C5	0.6 (3)
C7—C8—C9—C10	178.43 (14)	N—C1—C6—C5	177.43 (17)
C13—C12—C18—C21	111.27 (18)	F1—C3—C4—C5	179.11 (17)
C11—C12—C18—C21	−67.3 (2)	C2—C3—C4—C5	−1.6 (3)
C13—C12—C18—C20	−129.03 (17)	F2—C6—C5—C4	−179.41 (17)
C11—C12—C18—C20	52.4 (2)	C1—C6—C5—C4	−0.4 (3)
C13—C12—C18—C19	−9.4 (2)	C3—C4—C5—C6	0.8 (3)

### Hydrogen-bond geometry (Å, °)

**Table:**

*D*—H···*A*	*D*—H	H···*A*	*D*···*A*	*D*—H···*A*
O—H0···N	0.82	1.88	2.615 (2)	149
C16—H16A···O	0.96	2.27	2.935 (3)	126
C15—H15A···O	0.96	2.40	3.038 (2)	123
C7—H7···F1^i^	1.042	2.875	3.010 (2)	87
C16—H16C···N^i^	0.96	2.72	3.643 (3)	162
C21—H21B···F2^ii^	0.96	2.68	3.498 (3)	143

Symmetry codes: (i) *x*−1, *y*, *z*; (ii) −*x*+1/2, *y*+1/2, −*z*+1/2.
